# elearning for cancer immunotherapy

**DOI:** 10.3332/ecancer.2020.ed94

**Published:** 2020-01-16

**Authors:** Samuel L Hill, Peter WM Johnson

**Affiliations:** 1 Clinical Academic Research Fellow, Medical Oncology Registrar, University of Southampton, Centre for Cancer Immunology, University Hospital Southampton (MP 127), Tremona Road, Southampton, SO16 6YD, UK; 2 Professor of Medical Oncology, University of Southampton, Centre for Cancer Immunology, University Hospital Southampton (MP 127), Tremona Road, Southampton, SO16 6YD, UK

**Keywords:** immunotherapy, checkpoint inhibitors, elearning

## Abstract

Advances in cancer immunotherapy witnessed over the last decade with the licensing of numerous immune checkpoint inhibitors have greatly increased the application of this approach to treating advanced cancers. As a result, the number of health care professionals involved in the care of patients receiving immunotherapy treatments has grown. While the benefits can be significant, not all patients will experience them and toxicity can profound. elearning tools can help increase knowledge around the mechanisms, benefits and side effects of immunotherapies among clinical staff supporting patients undertaking such treatments.

## Introduction

The development of immune checkpoint inhibitor (ICI) therapy for advanced malignancies has changed the treatment landscape for a number of cancers, and the prospects for many patients over the past decade. The impressive outcomes observed has prompted significant expansion of research into immune-oncology treatments and the adoption of ICI as standard treatment for an expanding list of malignancies. Possessing idiosyncratic side effects not commonly seen by those accustomed to managing patients undergoing cytotoxic chemotherapy, the widespread adoption of ICI and other immunotherapies has created a need for reliable educational resources among those involved in looking after this expanding cohort of patients.

## Immune checkpoint inhibitors

The successes of ICI therapies has been well documented, with the progress made in treating unresectable malignant melanoma without doubt the highlight. This group of patients whose prognosis a decade ago was measured in only short months, were the first to benefit from such a therapeutic approach with the licensing of the anti-Cytotoxic T-lymphocyte-associated protein 4 (CTLA-4) monoclonal antibody ipilimumab in 2011 [1].

A treatment that demonstrated a response rate of over 20%, but most crucially, demonstrated durable responses after completion of treatment.

This success was shortly followed by inhibitory antibodies to Programmed cell death-1 (PD-1), Nivolumab and Pembrolizumab, which demonstrated further improvement in patient survival in the same cohort, even after anti-CTLA-4 treatment failure [2]. Combination therapy was the logical next step, producing yet further improvement in response rates and a median survival recently reported as in excess of 5 years [3].

Antibodies inhibiting PD-1 and its ligand (PD-L1) have also proven beneficial treating an expanding repertoire of diseases including non-small cell lung cancer, oropharyngeal squamous cell carcinoma, classical Hodgkin lymphoma and renal cell carcinoma among others. ICI therapy has been successfully combined with chemotherapy, and even shown benefit in settings considered poorly immunogenic such as triple negative breast cancer. These advances have been significant, but nowhere have the responses been as striking or durable as in the treatment of melanoma.

The first generation of ICIs are now being joined by a raft of other immune modulatory agents including those targeting other inhibitory receptors, either as mono-therapies, or more commonly in combinations. A study in 2017 identified in excess of 2000 immunotherapy agents under investigation, targeting 300 targets, and estimated over 3000 active clinical immune-oncology trials [4]. We can expect this number to be even greater today.

This rapidly changing environment can be increasingly difficult to navigate with new trial data being published weekly and licensing approvals almost as frequent. Oncologists seeking to provide optimal care for their patients require reliable and dynamic sources of information that can address their needs and adapt to a rapidly changing landscape. elearning modules such as those provided by *e*cancer can provide such educational resources [5].

Toxicity is also of crucial importance when considering the management of patients with ICIs, particularly in combinations such as ipilimumab and nivolumab where over 96% experience some treatment related side effects and 56% experienced hospitalising or life threatening grade 3 or 4 toxicity. Immune mediated diarrhoea and colitis, skin toxicity, pneumonitis and hepatitis, are commonly reported, as are endocrinopathies mediated by immune destruction of the thyroid, adrenal glands and pituitary [3]. When severe, management of these toxicities requires multidisciplinary input, which includes surgeons, gastroenterologist, endocrinologists and intensive care specialists among many others. While guidelines have been produced and published by international oncology organisations, these may not always be accessible to, or targeted at the expanding team detailed above. Open access elearning allows interested users to access relevant educational material in a flexible manner appropriate to their needs.

## Other immunotherapies

In addition to ICIs, there are a multitude of other approaches to harnessing an anti-cancer immune response in various stages of clinical development. Among the most high profile include Chimeric antigen receptor-T (CAR-T) lymphocyte technologies, which have demonstrated significant improvements in survival among individuals with relapsed and refractory haematological malignancies. Additionally, promising developments have been seen with personalised vaccine technologies, such as the licensing of dendritic cell vaccines in advanced prostate cancer, as well as experimental treatments utilising rapid sequencing technologies and identification of the tumour mutanome. Additionally viral therapies are making headway into clinical practice with the use of modified herpes viruses such as T-VEC.

As with ICIs treatments, the benefits of treatment are accompanied by significant toxicity. Patients receiving CAR-T treatments often require complex inpatient care, including input from intensive care specialists, among others, and is a further example of the extensive team involved in delivering the promise of cancer immunotherapies.

## A place for elearning

While it is clear the scope of cancer immunotherapy is increasingly far reaching and involves the collaboration of health professionals far beyond specialist oncology units, how does elearning in cancer medicine add value as an educational tool in for this target audience?

Firstly, elearning is not new to oncologists or those involved in cancer trials. Interactive computer based education has been routinely used to provide standardised training in research methodologies such as in accreditation in Good Clinical Practice for individuals engaging in clinical trials. Web-based elearning software has been demonstrated to be a valuable and effective tool in improving the clinical knowledge base of clinical trial investigators to ensure consistency across participating sites and countries [6]. Web-based distance learning techniques when evaluated have been shown to lead to improvements in, and retention of new knowledge, increased confidence in undertaking specific tasks, and often have a high completion rate. The same education platform can be used across specialties and professions [6].

elearning has proven to be an effective tool in post-graduate medical training [7], offering a flexible and convenient approach to education across many subjects [8, 9]. This approach has been encouraged by many national health organisations such as the United Kingdom’s Department of Health [10].

The elearning resources presented by *e*cancer are not the only online educational resources about cancer immunotherapy, but do provide a broad curriculum of topics that would be of interest to oncologists and other health care professionals. The course is open access, free and can be approached in a way that is adaptable to the needs of the user.

## Conclusion

The licencing of immune checkpoint inhibitors for use in the treatment of advanced melanoma, as well as a number of other malignancies, has seen the reach of immunotherapy increase significantly. Other immunotherapeutic strategies such as CAR-T cell therapy are also starting to make an impact in the clinic. elearning resources can help healthcare professionals involved in the care of patients with advanced cancer develop their knowledge and understanding of these exciting novel treatments.

## Conflicts of interest

**Samuel L Hill:** no conflicts of interest; receives funding via Cancer Research UK.

**Peter WM Johnson:** Advisory Boards/honoraria: Takeda, Bristol-Myers Squibb, Novartis, Celgene, Kite pharma, Epizyme, Genmab, Incyte, Morphosys, Kymera, Janssen, Oncimmune.

## Funding statement

No funding was received for the writing and publication of this article.
